# Diferencias relacionadas al sexo en pacientes con infarto agudo de *Miocardio ST Elevado*

**DOI:** 10.47487/apcyccv.v1i1.10

**Published:** 2020-03-30

**Authors:** Francisco Chávez, Sandra Espinola, Manuel Chacón

**Affiliations:** 1 Médico residente de Cardiología - Instituto Nacional Cardiovascular INCOR. Lima, Perú. Instituto Nacional Cardiovascular INCOR Lima Perú; 2 Servicio de Cardiología Clínica - Instituto Nacional Cardiovascular INCOR. Lima, Perú. Servicio de Cardiología Clínica Instituto Nacional Cardiovascular INCOR Lima Perú

**Keywords:** infarto de miocardio, sexo, mortalidad, myocardial infarction, sex, mortality

## Abstract

**Objetivo:**

Determinar las características epidemiológicas, localización del infarto, tipo y tiempos de reperfusión, así como los eventos adversos intrahospitalarios, distribuidos por sexo en pacientes con infarto de miocardio con elevación del segmento ST (IAMCEST) en Perú.

**Métodos:**

Es un sub-análisis del PEruvian Registry of ST-segment Elevation Myocardial Infarction (PERSTEMI), el cual fue un estudio observacional, prospectivo y multicéntrico de pacientes mayores de 18 años que fueron hospitalizados por infarto de miocardio con elevación del segmento ST. Se compararon las características epidemiológicas, clínicas, manejo y eventos adversos intrahospitalarios según sexo.

**Resultados:**

De los 396 pacientes, se encontró un 20.9% de pacientes mujeres, con un predominio de población octogenaria sobre los hombres. La hipertensión arterial fue el factor de riesgo más frecuente en el sexo femenino (74.7 Vs. 50%, p = 0.001); así como como las manifestaciones clínicas atípicas como disnea (40.9 Vs. 27.1%, p = 0.012) y síncope (10.8 vs. 3.8%, p = 0.017). Por otro lado, la localización del infarto de miocardio en cara inferior fue más frecuente en mujeres (51.8 vs. 38.98%). En ambos sexos, no hubo diferencias significativas respecto a la terapia de reperfusión empleada (Fibrinólisis, ICP primaria, ICP en general); así como en los tiempos de isquemia (6 vs. 5.6 horas, p = 0.456), tiempos de reperfusión y estancia hospitalaria. Sin embargo, el sexo femenino presentó mayor mortalidad intrahospitalaria (21.6 vs. 7%, p = 0.001), complicaciones mecánicas (8.4 vs. 1.9%, p = 0.008), shock cardiogénico (15.6 vs. 9.5%, p = 0.087) e insuficiencia cardiaca (33.7 vs 24.9%, p = 0.072).

**Conclusiones:**

El IAMCEST en el sexo femenino se presenta a una edad significativamente mayor en comparación con los varones y está asociado a mayor mortalidad intrahospitalaria y complicaciones mecánicas.

## Abstract (en)

**Objective:**

To determine the epidemiological characteristics, location of the infarction, type and times of reperfusion, as well as in-hospital adverse events, distributed by sex in patients with ST-segment elevation myocardial infarction (STEMI) in Peru.

**Methods:**

It is a sub-analysis of the PEruvian Registry of ST-segment Elevation Myocardial Infarction (PERSTEMI), which was an observational, prospective and multicenter study about patients over 18 years-old, who were hospitalized for ST-segment elevation myocardial infarction. Epidemiological and clinical characteristics, management and in-hospital adverse events were compared according to sex.

**Results:**

396 patients were studied, 20.9% were female, with a predominance of octogenarian population over men. High blood pressure was the most frequent risk factor in women (74.7 Vs. 50%, p = 0.001); as well as atypical clinical manifestations such as dyspnea (40.9 Vs. 27.1%, p = 0.012) and syncope (10.8 vs. 3.8%, p = 0.017). On the other hand, the inferior wall myocardial infarction was more frequent in women (51.8 vs. 38.98%). There were no significant differences regarding the reperfusion therapy used (Fibrinolysis, primary PCI, PCI in general); as well as in times of ischemia (6 vs. 5.6 hours, p = 0.456), reperfusion times and hospital stay between both sexes. However, the female sex presented higher in-hospital mortality (21.6 vs. 7%, p = 0.001), mechanical complications (8.4 vs. 1.9%, p = 0.008), cardiogenic shock (15.6 vs. 9.5%, p= 0.087) and heart failure (33.7 vs. 24.9%, p = 0.072).

**Conclusions:**

STEMI in females presents at significantly older age compared to males and is associated with higher in-hospital mortality and mechanical complications.

Actualmente, la enfermedad cardiovascular es la primera causa de morbilidad y mortalidad en las mujeres^1^ Varios estudios han demostrado diferencias basada en el sexo respecto a los factores de riesgo, las comorbilidades, la anatomía coronaria, el cuadro clínico inicial, la eficacia del tratamiento y los resultados del síndrome coronario agudo (SCA). ^(^^2^ Esta discrepancia tiene origen multifactorial; las mujeres presentan enfermedad coronaria a una edad más tardía, con mayor carga de comorbilidades, probabilidad más alta de presentar síntomas atípicos, lo que origina retraso en el contacto con una unidad de emergencia. ^(^^3^^,^^4^ La evidencia sugiere que después de un SCA las mujeres presentan con más frecuencia complicaciones, re-hospitalización y mayor riego de mortalidad. ^(^^2^

El infarto de miocardio con elevación del segmento ST (IAMCEST), es una de las causas principales de mortalidad en hombres y mujeres. A nivel nacional no existen estudios que describan las características de IAMCEST en mujeres. Por este motivo, se realizó un sub-análisis descriptivo a partir del registro PERSTEMI sobre las características del IAMCEST en mujeres en comparación con hombres, tratados desde febrero de 2016 a febrero de 2017 a fin de conocer su comportamiento, distribución y así poder contribuir en la mejora de la conducta terapéutica, el pronóstico y los resultados.

## Material y Método

Se trata de un sub-análisis del PEruvian Registry of ST-segment Elevation Myocardial Infarction (PERSTEMI), ^(^^5^ el cual fue un estudio observacional, prospectivo y multicéntrico; que incluyó a veinte centros hospitalarios del Perú (Ministerio de Salud, EsSalud, fuerzas armadas y clínicas privadas). Los criterios de inclusión y exclusión del estudio fueron previamente publicados. ^(^^5^ Este sub-análisis comparó las características epidemiológicas, localización del infarto, tipo y tiempos de reperfusión, así como los eventos adversos que se presentaron durante la hospitalización según el sexo del paciente.

El análisis de las variables cualitativas se realizó utilizando el estadístico chi-cuadrado o el test de Fisher. Para la evaluación de la normalidad en las variables cuantitativas se utilizó el test de Shapiro-Wilk; y se tomó la media y la desviación estándar para el análisis de variables con distribución normal, caso contrario, se utilizó la mediana y el rango intercuartil (RIQ). Además, se realizó análisis estratificado de Mantel-Haenszel para análisis del grupo etáreo y la mortalidad intrahospitalaria.

## Resultados

En el estudio PERSTEMI se incluyeron a 396 pacientes con diagnóstico de IAMCEST entre febrero del 2016 a febrero del 2017, las características basales según sexo se encuentran en la Tabla 1. Del total, 83 fueron mujeres (21%) y 313 (79%) hombres. La edad media al ingreso hospitalario fue mayor en las mujeres que en los hombres. Por otro lado, las mujeres comparadas a los hombres tuvieron mayor porcentaje de población octogenaria (37.3 Vs. 7.6%).

**Tabla 1:** Características basales de la población del registro PERSTEMI según sexo

	Mujer (n=83)		Varón (n=313)		p
Edad (años)	73.6	(±10.9)	62.5	(±11.7)	0.0001
Antecedentes patológicos					
Hipertensión arterial	62	(74.7)	159	(50.0)	0.001
Diabetes mellitus	24	(28.9)	72	(23.0)	0.264
Dislipidemia	31	(37.3)	107	(34.1)	0.652
Enfermedad renal crónica	5	(6.0)	7	(2.2)	0.08
Síntomas de Inicio					
Angina	67	(80.0)	290	(92.6)	0.003
Disnea	34	(40.9)	85	(27.1)	0.012
Dolor torácico atípico	6	(7.2)	16	(5.1)	0.427
Síncope	9	(10.8)	12	(3.8)	0.017
Clasificación clínica al ingreso					0.178
Killip - Kimball I	52	(62.6)	210	(67)	
Killip - Kimball II	21	(25.3)	82	(26.2)	
Killip - Kimball III	6	(7.2)	7	(2.2)	
Killip - Kimball IV	4	(4.8)	14	(4.4)	
Localización					0.09
Anterior	39	(47.0)	188	(60.1)	
Inferior	43	(51.8)	122	(39.0)	
Lateral	1	(1.2)	3	(1.0)	
Reperfusión					
Sí	66	(79.5)	266	(84.9)	0.242
No	17	(20.4)	47	(15.0)	
Fibrinólisis	29	(34.9)	130	(41.5)	
Intervención coronaria percutánea primaria	29	(34.9)	87	(27.8)	
Eventos adversos durante la hospitalización					
Muerte intrahospitalaria	18	(21.6)	22	(7.0)	0.001
Complicación mecánica	7	(8.4)	6	(1.9)	0.008
Choque cardiogénico	13	(15.6)	30	(9.5)	0.087
Insuficiencia cardíaca	28	(33.7)	78	(24.9)	0.072

Se reporta medias (desviación estándar) y frecuencias (porcentaje) para variables cuantitativas y categóricas, respectivamente.

Entre los factores de riesgo cardiovascular, se evidenció una mayor frecuencia de mujeres con hipertensión arterial, estando presente en más de la mitad de pacientes; mientras que diabetes mellitus, dislipidemia y enfermedad renal crónica, fueron de presentación similar en ambos sexos. Entre los síntomas de presentación, la angina fue el síntoma predominante en mujeres, pero fue menos frecuente que en los hombres. Por otro lado, la disnea y el síncope como síntoma inicial, se presentaron con predominancia en mujeres.

La localización más frecuente del infarto en mujeres fue de la cara inferior en más de la mitad de los casos, seguido de la localización anterior y lateral; a diferencia de los hombres en donde la localización más frecuente fue anterior (en tres quintas partes de los pacientes), luego inferior y finalmente lateral. Asimismo, al evaluar la presentación del infarto según la clasificación de Killip-Kimball, se evidenció que la clase I fue la más frecuente en ambos sexos, sin diferencias significativas entre ambos ellos. 

### Reperfusión

Se evidenció que las mujeres representaron el 19.8% del total de pacientes que recibieron alguna terapia de reperfusión, la cual se realizó en el 83.8% del total de los pacientes del registro PERSTEMI. La fibrinólisis, ICP (intervención coronaria percutánea) primaria e ICP en general se realizaron de forma similar en mujeres y hombres.

### Eventos adversos durante la hospitalización 

**Figura 1:**
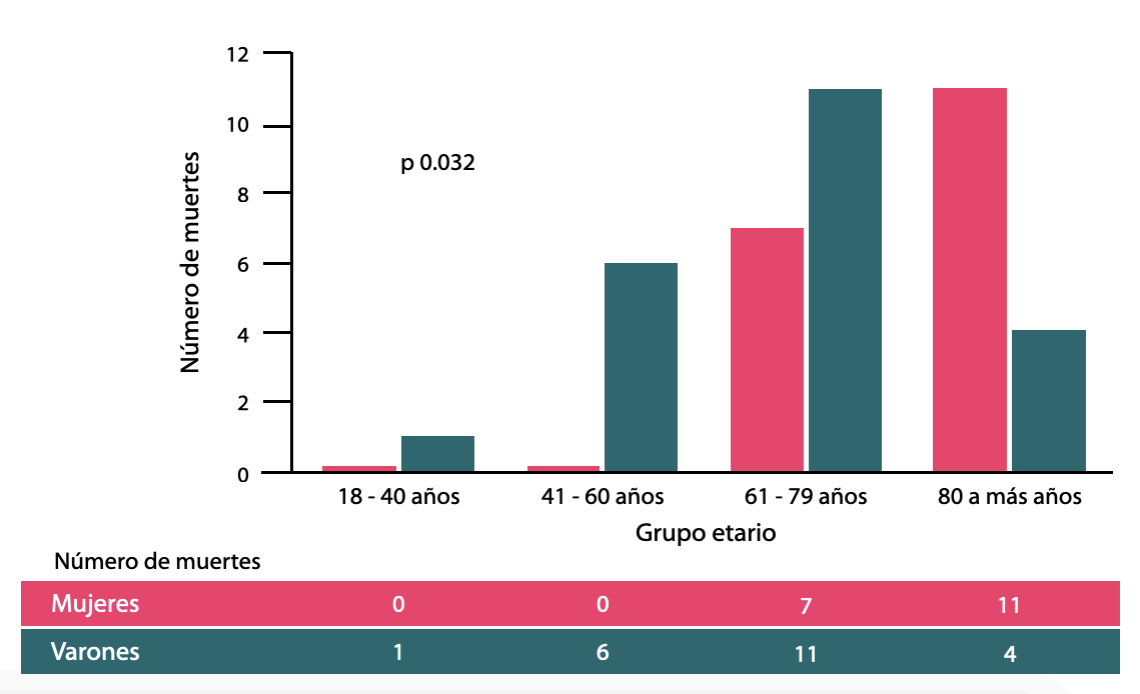
Mortalidad Intrahospitalaria según sexo y grupo etario.

En nuestro estudio, la mortalidad intrahospitalaria fue mayor en mujeres que en varones, siendo el sexo femenino un factor relacionado a incremento del riesgo de mortalidad (OR: 3.6, IC 95%: 1.8-7.2, p= 0.0001), y complicación mecánica (OR: 4.7, IC 95%: 1.5-14.4, p= 0.007), hallazgos que podrían estar en relación a la edad de presentación más avanzada observada en las mujeres (Figura 1). Por otro lado, no hubo diferencias en la ocurrencia de sangrado en general (mujeres 3.6%, varones 4.5%, p=0.732), ni en la de sangrado mayor (mujeres 1.2%, varones 0.6%, p=0.597).

### Tiempos

La fibrinólisis y la ICP primaria, se realizaron de forma similar en mujeres y hombres (Tabla 1), aunque el tiempo a lisis y el tiempo a ICP (Tabla 2) estuvieron igualmente prolongados; esto debido a que un gran número de pacientes se presentaban a centros sin capacidad de ICP, y la demora en transferencia aumentó el tiempo de isquemia esperado. Por otro lado, la estancia hospitalaria promedio no superó la semana (Tabla 2) y no tuvo diferencias respecto al sexo.

**Tabla 2:** Tiempos hasta la reperfusión y estancia hospitalaria

	Mujer (n=83)		Varón (n=313)		p
Tiempo hasta la reperfusión (horas)					
Tiempo de isquemia	6	(4 - 9)	5.6	(3.5 - 11)	0.456
Tiempo a lisis	3.5	(2.5 - 6)	4	(3 - 5.8)	0.969
Tiempo a intervención coronaria percutánea	8	(6 - 11)	8	(5 - 14)	0.431
Estancia hospitalaria (días)					
Tiempo de estancia hospitalaria	5	(4 - 8)	7	(5 - 10)	0.103

Se reporta mediana (y rango intercuartil) para cada variable.

## Discusión

Este es el primer análisis en el Perú enfocado sobre el comportamiento del IAMCEST en mujeres, en base a los datos del registro PERSTEMI. Los principales hallazgos de nuestro estudio fueron, que las mujeres se presentan a una edad significativamente mayor que los hombres, lo cual guarda relación con un registro francés; ^(^^4^ por otro lado, según este mismo registro, se reporta una disminución de la prevalencia de 3.6% del IAMCEST en mujeres octogenarias desde el 2004 al 2014, sin embargo, en nuestro estudio la población octogenaria tuvo una predominancia en el sexo femenino, y no puede ser comparada con registros nacionales previos pues es el primero reportado en este grupo etario.

Un registro canadiense sobre SCA, ^(^^3^ encontró que las mujeres tenían mayor prevalencia de hipertensión arterial, diabetes mellitus, dislipidemia, accidente cerebrovascular previo, y enfermedad vascular periférica; así como en el registro ISACS-TC^6^ donde la presencia de diabetes mellitus, hipertensión arterial y antecedente familiar de cardiopatía isquémica fueron mayormente reportadas en mujeres; nosotros evidenciamos la predominancia sólo de hipertensión arterial en el sexo femenino. La presencia de síntomas atípicos también ha sido reportada con mayor frecuencia en mujeres, ^(^^7^^,^^8^ de la misma manera, en nuestro estudio se encontró que, si bien la angina fue el síntoma de presentación más frecuente en ambos sexos, la disnea y el síncope se manifestaron con mayor frecuencia en mujeres, lo cual mantiene la tendencia de presentación de síntomas atípicos en el sexo femenino en nuestro medio.

Comparado al registro ISACS-TC, ^(^^6^ según la clasificación de Killip-Kimball hubo una predominancia de la clase I y una frecuencia decreciente según la progresión de clase, sin diferencias significativas entre ambos sexos, de forma similar a lo que se encontró en el presente estudio; sin embargo, la localización del IAMCEST en cara inferior fue lo más frecuente, siendo discordante con el registro mencionado.

En el registro NCVD-ACS, ^(^^9^ una proporción significativamente menor de mujeres recibió intervención coronaria percutánea primaria (6.2% vs. 6.7%, respectivamente, p = 0.001) y fibrinólisis (64.4% vs. 74.6%, respectivamente, p = 0.000), algo que no encontramos en este estudio; aunque el tiempo a lisis y el tiempo a ICP fue igualmente mayor al recomendado por las guías internacionales; ^(^^10^^,^^11^ lo cual estuvo en relación al gran número de pacientes que acudieron a centros sin capacidad de ICP y la demora en la transferencia hacia centros con ICP, incrementando de tal modo el tiempo de isquemia.

La mayor mortalidad intrahospitalaria reportada en el sexo femenino, podría estar en relación con mayores complicaciones mecánicas, la localización del infarto y edad de presentación más avanzada. ^(^^12^ Existe variación de la localización del IAMCEST relacionada con las diferentes complicaciones mecánicas: el IAMCEST de la pared infero-lateral y posterior se asocia con mayor frecuencia a ruptura de musculo papilar, que a su vez condiciona insuficiencia mitral aguda severa y a defecto del septum ventricular posterior, el cual comparado con el defecto ventricular anterior, tiene peor pronóstico porque es más complejo en morfología y frecuentemente se asocia con disfunción ventricular derecha importante. ^(^^13^^,^^14^ Así mismo, Elbadawi A et al. ^(^^15^ evidenciaron que pacientes con IAMCEST y complicaciones mecánicas presentaron una tasa de mortalidad hospitalaria del 42.4% en comparación con el 12.7% de mortalidad en pacientes sin complicaciones mecánicas. Por otro lado, el registro ACC-NCDR^16^ evidenció que las mujeres tenían tasas más altas de shock cardiogénico e insuficiencia cardíaca congestiva. Es por ello, que en este sub-análisis, se propone que la mayor mortalidad intrahospitalaria evidenciada en las mujeres se podría sustentar en la edad de presentación más avanzada, mayor número de IAMCEST de pared inferior y consiguiente mayor número de complicaciones mecánicas, a pesar de no haber diferencias según sexo en los tiempos de isquemia, sangrado, ni en la ocurrencia de falla cardiaca y shock cardiogénico. 

Así mismo, Poon et al. ^(^^17^ en Canadá, evidenciaron que el sexo femenino se asoció a una mayor mortalidad hospitalaria (OR ajustado 1.26; IC 95%: 1.02- 1.56, p = 0.036), independientemente de la edad (p de interacción = 0.76). Sin embargo, Lu et al. ^(^^9^ evidenciaron que las mujeres tenían una mortalidad hospitalaria no ajustada significativamente más alta que los hombres (15.0% vs. 8.1%, respectivamente, p <0.001); pero, después de ajustar las diferencias de edad y otras covariables, la mortalidad intrahospitalaria no fue significativamente diferente entre mujeres y hombres (OR ajustado: 1.06, IC 95%: 0.67 a 1.70, p = 0.769). Esta diferencia se podría explicar por el promedio de edad menor en las mujeres que participaron en el registro NCVD-ACS comparado con nuestro estudio.

Este estudio presenta la limitación de tomar los datos de un registro nacional, que tuvo la mayor cantidad de sus pacientes concentrados en la capital y en hospitales docentes, lo cual podría no ser representativo de la realidad de nuestro país.

## Conclusiones

En este subestudio del PERSTEMI, las mujeres que presentan un IAMCEST, lo hacen a una edad significativamente mayor que los hombres, y tienen la HTA como principal factor de riesgo asociado. La cara inferior es la más frecuentemente afectada, no encontramos diferencias significativas entre las estrategias de reperfusión, pero sí en la ocurrencia de más eventos adversos (muerte intrahospitalaria y complicaciones mecánicas) comparado a los hombres.

## Financiación:

Ninguna

## References

[1] Sarma A, Braunwald E, Cannon C (2019). Outcomes of Women Compared with Men After Non-ST-Segment Elevation Acute Coronary Syndromes. J Am Coll Cardiol.

[2] Graham G (2016). Acute Coronary Syndromes in Women Recent Treatment Trends and Outcomes. Clinical Medicine Insights: Cardiology.

[3] Udell J, Koh M, Qiu F (2017). Outcomes of Women and Men with Acute Coronary Syndrome Treated with and without Percutaneous Coronary Revascularization. J Am Heart Assoc.

[4] Gabet A, Danchin N, Juilliere Y (2017). Acute coronary syndrome in women: rising hospitalizations in middle-aged French women, 2004-14. European Heart Journal.

[5] Chacón M, Vega A, Aráoz O (2018). Características epidemiológicas del infarto de miocardio con elevación del segmento ST en Perú resultados del PEruvian Registry of ST-segment Elevation Myocardial Infarction (PERSTEMI). (2018). Arch Cardiol Mex.

[6] Cenko E, Van der Schaar M, Yoon J (2019). Sex-Related Differences in Heart Failure After ST-Segment Elevation Myocardial Infarction. J Am Coll Cardiol.

[7] McSweeney J, Rosenfeld A, Abel W (2016). Preventing and experiencing ischemic heart disease as a woman state of the science: a scientific statement from the American Heart Association. Circulation.

[8] Bairey C, Shaw L, Reis S (2006). In-sights from the NHLBI-Sponsored Women's Ischemia Syndrome Evaluation (WISE) Study Part II: gender differences in presentation, diagnosis, and outcome with regard to gender-based pathophysiology of atherosclerosis and macrovascular and microvascular coronary disease. J Am Coll Cardiol.

[9] Lu H, Nordin R, Wan W (2014). Sex Differences in Acute Coronary Syndrome in a Multiethnic Asian Population Results of the Malaysian National Cardiovascular Disease Database-Acute Coronary Syndrome (NCVD-ACS) Registry. Glob Heart.

[10] Wong G, Welsford M, Ainsworth C (2019). 2019 Canadian Cardiovascular Society / Canadian Association of Interventional Cardiology Guidelines on the Acute Management of STE-Elevation Myocardial Infarction Focused Update on Regionalization and Reperfusion. Canadian Journal of Cardiology.

[11] Ibañez B, James S, Agewall S (2017). Guía ESC 2017 sobre el tratamiento del infarto agudo de miocardio en pacientes con elevación del segmento ST. Rev Esp Cardiol.

[12] Birnbaum Y, Fishbein M, Blanche C (2002). Ventricular Septal Rupture after Acute Myocardial Infarction. N Engl J Med.

[13] Durko A, Budde R, Geleijnse M (2018). Recognition, assessment and management of the mechanical complications of acute myocardial infarction. Heart.

[14] Caballero J, Hernández J, Sanchis J (2009). Complicaciones mecánicas en el infarto agudo de miocardio. ¿Cuáles son, cuál es su tratamiento y qué papel tiene el intervencionismo percutáneo?. Rev Esp Cardiol Supl.

[15] Elbadawi A, Elgendy I, Mahmoud K (2019). Temporal trends and outcomes of mechanical complications in patients with acute myocardial infarction. JACC Cardiovasc Interv.

[16] Akhter N, Milford S, Roe M (2009). Gender differences among patients with acute coronary syndromes undergoing percutaneous coronary intervention in the American College of Cardiology-National Cardiovascular Data Registry. Am Heart J.

[17] Poon S, Goodman S, Yan R (2012). Bridging the gender gap insights from a contemporary analysis of sex-related differences in the treatment and outcomes of patients with acute coronary syndromes. Am Heart J.

